# Rescue catheter directed treatment for floating right atrial thrombi and deep vein thrombosis in patient with high-risk acute pulmonary embolism: a case report of multidisciplinary approach

**DOI:** 10.1093/ehjcr/ytaf559

**Published:** 2025-11-04

**Authors:** Filippo Russo, Gianluca Ricchetti, Silvia Ajello, Domenico Baccellieri, Luca Angelo Ferri, Beatrice Peveri, Anna Mara Scandroglio, Matteo Montorfano

**Affiliations:** Department of Interventional Cardiology, IRCCS San Raffaele Scientific Institute, Via Olgettina 60, 20132 Milan, Italy; Department of Interventional Cardiology, IRCCS San Raffaele Scientific Institute, Via Olgettina 60, 20132 Milan, Italy; Cardiac Intensive Care Unit, Cardio-Thoracic-Vascular Department, IRCCS San Raffaele Scientific Institute, Via Olgettina 60, 20132 Milan, Italy; Vein Center, Vascular Surgery Unit, Cardio-Thoracic-Vascular Department, IRCCS San Raffaele Scientific Institute, Via Olgettina 60, 20132 Milan, Italy; Department of Interventional Cardiology, IRCCS San Raffaele Scientific Institute, Via Olgettina 60, 20132 Milan, Italy; Cardiac Intensive Care Unit, Cardio-Thoracic-Vascular Department, IRCCS San Raffaele Scientific Institute, Via Olgettina 60, 20132 Milan, Italy; Cardiac Intensive Care Unit, Cardio-Thoracic-Vascular Department, IRCCS San Raffaele Scientific Institute, Via Olgettina 60, 20132 Milan, Italy; Department of Interventional Cardiology, IRCCS San Raffaele Scientific Institute, Via Olgettina 60, 20132 Milan, Italy; School of Medicine, Vita-Salute San Raffaele University, Via Olgettina 58, 20132 Milan, Italy

**Keywords:** Right atrial thrombi, Pulmonary embolism, Deep vein thrombosis, Catheter-directed treatment, Mechanical thromboaspiration, Case report

## Abstract

**Background:**

High-risk pulmonary embolism (PE) complicated by floating right atrial thrombi (RAT) carries a significantly increased mortality risk, often requiring urgent intervention beyond systemic thrombolysis (ST). However, current guidelines lack specific recommendations for managing persistent RAT following ST. Catheter-directed therapies (CDTs) have emerged as a promising alternative in this setting. We report a novel single-session, multidisciplinary approach combining mechanical thrombectomy of both RAT and iliofemoral deep vein thrombosis (DVT) using the Penumbra Indigo Lightning System.

**Case summary:**

A 74-year-old woman presented with syncope and signs of cardiogenic shock. Imaging confirmed high-risk PE with floating RAT and extensive iliofemoral DVT. Initial treatment with ST led to partial haemodynamic recovery but persistent RAT. A multidisciplinary Pulmonary Embolism Response Team elected to perform percutaneous thrombectomy. Under transoesophageal echocardiography and fluoroscopic guidance, mechanical aspiration of the right atrial thrombus was successfully performed using the Penumbra Indigo Lightning 12 F catheter. In the same session, intravascular ultrasound-guided aspiration of iliofemoral DVT was achieved, with confirmation of complete thrombus removal and exclusion of the May–Thurner syndrome. The patient had an uneventful recovery, with restored right ventricular function and no recurrent thromboembolism or post-thrombotic syndrome at follow-up.

**Discussion:**

This case demonstrates the feasibility and safety of a combined single-session catheter-directed approach for simultaneous treatment of RAT and DVT following ST failure. The integration of multidisciplinary decision-making, advanced aspiration technology, and intravascular imaging may optimize outcomes in complex PE cases and supports expanding the role of CDTs in rescue settings.

Learning points

- Mechanical thrombectomy using the Penumbra Indigo Lightning System can safely and effectively remove right atrial and iliofemoral thrombi in high-risk pulmonary embolism (PE) patients, even after systemic thrombolysis.
- A multidisciplinary Pulmonary Embolism Response Team is essential for timely decision-making and coordinated treatment in complex PE cases.

## Introduction

According to the 2019 European Society of Cardiology (ESC) Guidelines on acute pulmonary embolism (PE), patient’s risk stratification is highly recommended to guide appropriate therapeutic management.^1^ In this context, high-risk PE defined according to the ESC Guidelines is frequently associated with poor clinical outcomes despite prompt diagnosis and treatment, with short-term mortality rates ranging from 30% to 50%. In this clinical setting, concomitant floating right atrial thrombi (RAT) represent a rare but life-threatening condition associated with mortality rate approaching 90%.^2–4^ Despite their prognostic implication, current Guidelines do not incorporate RAT into existing risk stratification models, and evidence remains scarce regarding the use of advanced therapies, such as catheter-directed treatments (CDTs), in high-risk PE. As a result, systemic thrombolysis (ST) remains the first-line therapy, despite its relatively high failure rate (nearly 10%) and the frequent presence of contraindications in this population.^5^ Furthermore, ST failure, defined as persistent clinical instability and residual right ventricular (RV) dysfunction within the first 36 h, represents a critical and potentially fatal scenario, and when concomitant with PE, the absence of RAT resolution after ST also constitutes a treatment failure, given the persistence of a potentially lethal condition despite haemodynamic improvement due to possible recurrence.^6^ Historically, the only available bail-out strategy in these scenarios was represented by surgical thrombo-embolectomy; in recent years, CDTs, with large-bore percutaneous aspiration catheters, gained favour for the removal of RAT.^7–9^ Nonetheless, limited evidence exists regarding newer generation and smaller percutaneous catheters, such as the Penumbra Indigo Lightning System (Penumbra, Alameda, CA, USA).^10,11^ Given the complexity and multiple comorbidities of PE patients, a multidisciplinary approach—integrating various specialists within a Pulmonary Embolism Response Team (PERT)—has become essential.^12,13^ This case illustrates a novel multidisciplinary strategy for managing high-risk PE with concurrent floating RAT and extensive iliofemoral deep vein thrombosis (DVT). Here, ST was effective in patient’s haemodynamic recovery but failed to solve RAT, requiring aspiration thrombectomy by means of the Penumbra Indigo Lightning System®. Furthermore, the source of embolization in the iliofemoral venous was simultaneously addressed with the same device in a single combined procedure performed by an interventional cardiologist and vascular surgeon.

## Summary figure

**Table:** 

**10 Days prior admission**	The patient experienced exertional dyspnea, fatigue and concomitant left lower limb swelling.
**2 Hours prior admission**	Syncopal episode with cranial trauma. Worsening of dyspnea.
**At admission (Day 0)**	Hemodynamic deterioration associated with echocardiographic evidence of floating right heart thrombi. CT Pulmonary Angiography (CTPA): Bilateral pulmonary embolism with right atrial floating thrombus. Lower extremity ultrasound: extensive left iliofemoral DVT.
**30 minutes**	Bed-side emergent thrombolysis (rTPA) with hemodynamic improvement and admission to cardiac ICU unit.
**1 hour**	Persistence of floating Right Atrial Thrombi. Multidisciplinary discussion in PERT team leading to decision of Catheter Directed Mechanical Thrombectomy.
**2 Hours**	Right atrial thrombi removed with percutaneous thrombectomy via Penumbra Indigo Lightning system. Iliofemoral DVT treated in the same session with percutaneous thrombectomy. Post-thrombectomy intravascular ultrasound (IVUS) of ilio-femoro-popliteal axis confirmed complete thrombus removal. Atrial fibrillation treated with electric cardioversion.
**Day 2–4**	Progressive weaning from inotropic and ventilatory support.
**Day 10**	Pre-discharge echocardiography showing right ventricular function recovery with persistence of mild right ventricle dilatation. Persistence of mild pulmonary artery hypertension.
**Month 1**	Complete recovery of right ventricular function with normalization of pulmonary pressures. No evidence of recurrent embolism or post-thrombotic syndrome.

## Case presentation

**Figure 1:**
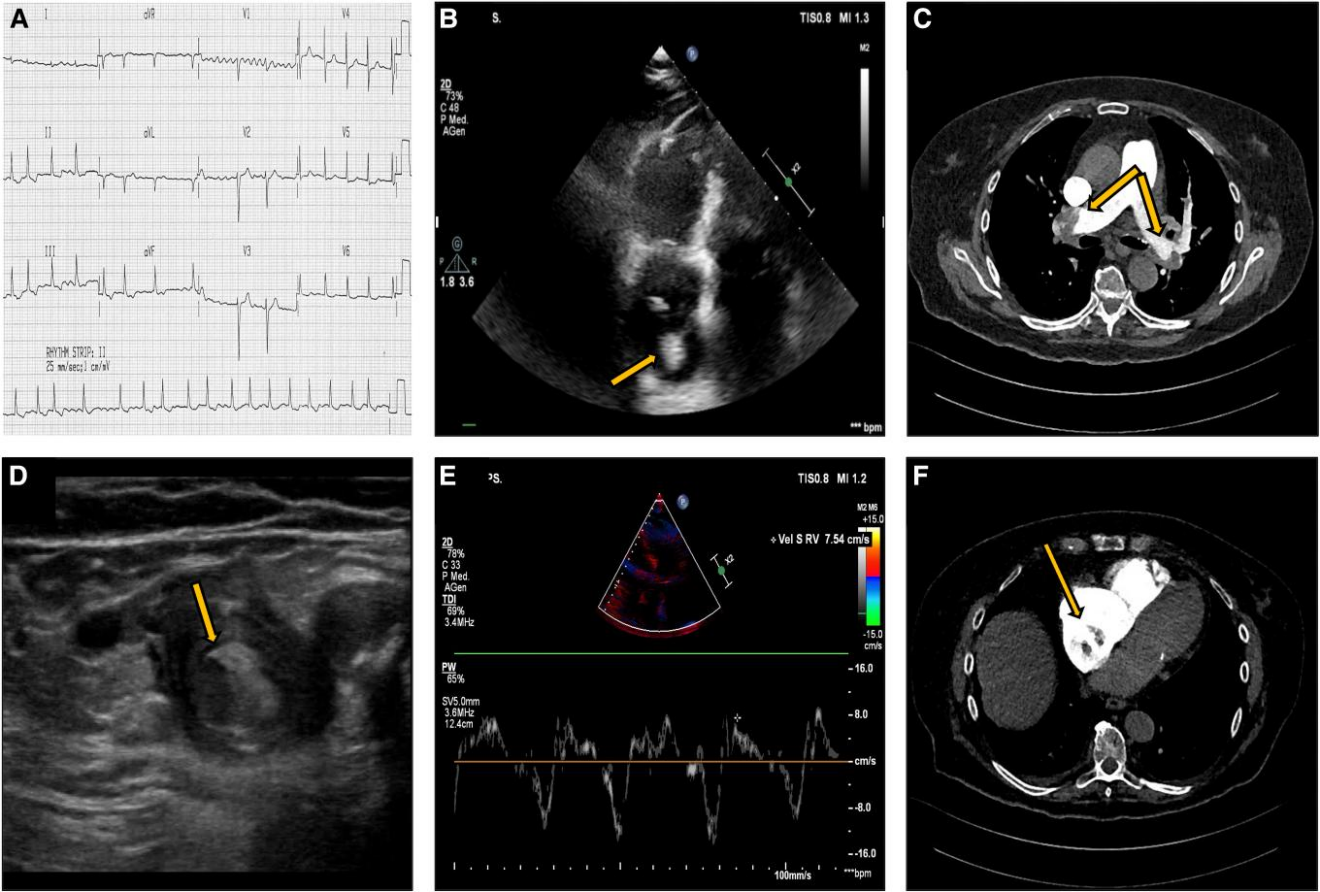
Characteristics at presentation. (*A*) Electrocardiogram with atrial fibrillation and rapid ventricular response. (*B*) Floating thrombus in the right atrium on transthoracic echocardiography (arrow). (*E*) Tissue Doppler imaging of the right ventricle consistent with significant right ventricular dysfunction. (*C* and *F*) CT pulmonary angiography revealing bilateral pulmonary embolism with thrombotic occlusion of the main branches of the pulmonary arteries and evidence of a right atrial thrombus (arrow). (*D*) Lower limb ultrasound identifying a deep vein thrombosis in the popliteal vein (arrow).

**Figure 2:**
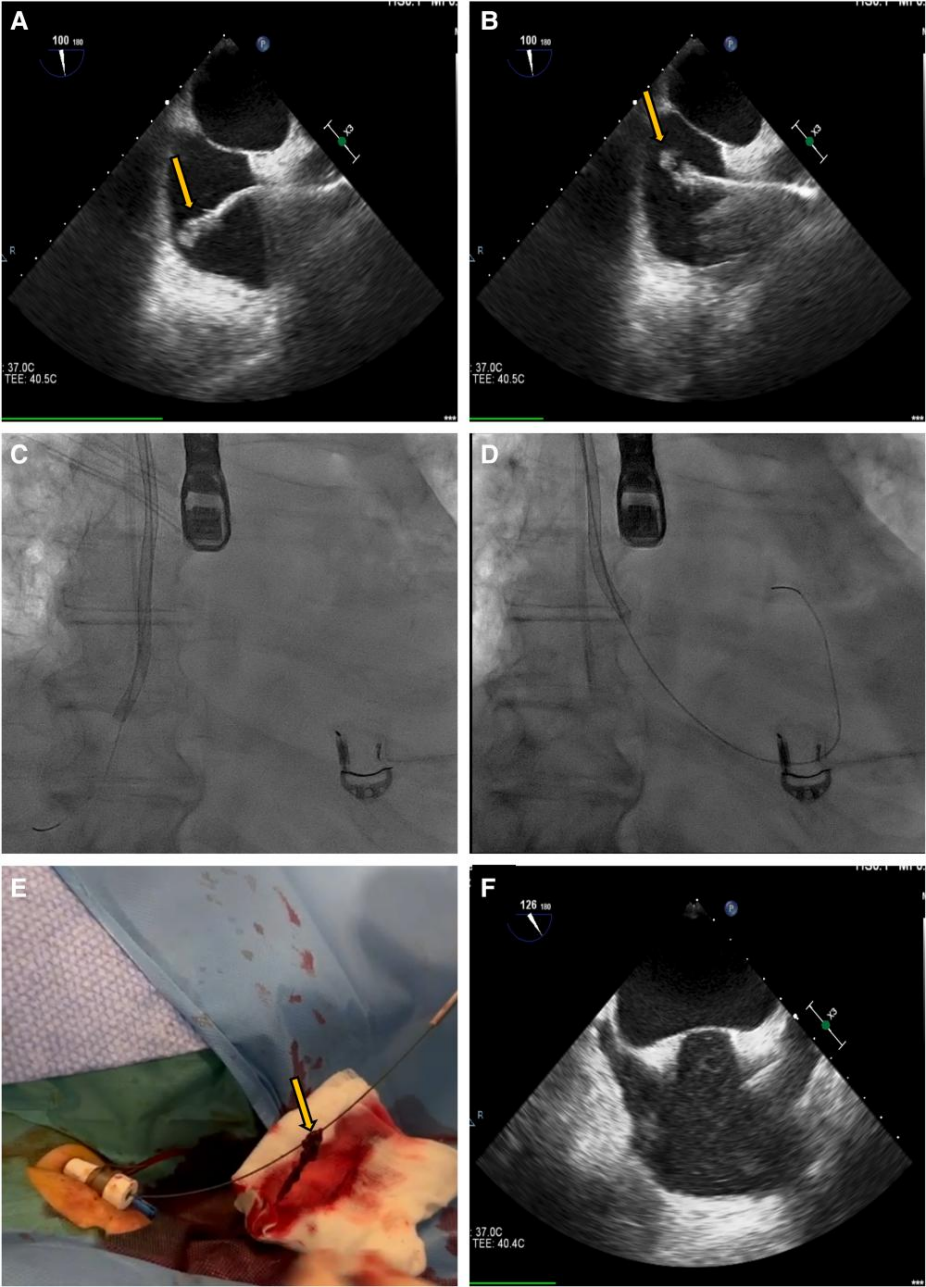
Intraprocedural characteristics. (*A* and *B*) Transoesophageal echocardiography-guided aspiration of the right atrial thrombus using the Penumbra Indigo Lightning System (arrow). (*C* and *D*) Fluoroscopic guidance of thrombus aspiration with the Penumbra catheter advanced over a 0.032′ Storq guidewire used as a separator. (*E*) The extracted thrombotic material (arrow). (*F*) The complete removal of the right atrial thrombus following catheter-directed therapy, as demonstrated by the absence of residual thrombus on echocardiography.

**Figure 3:**
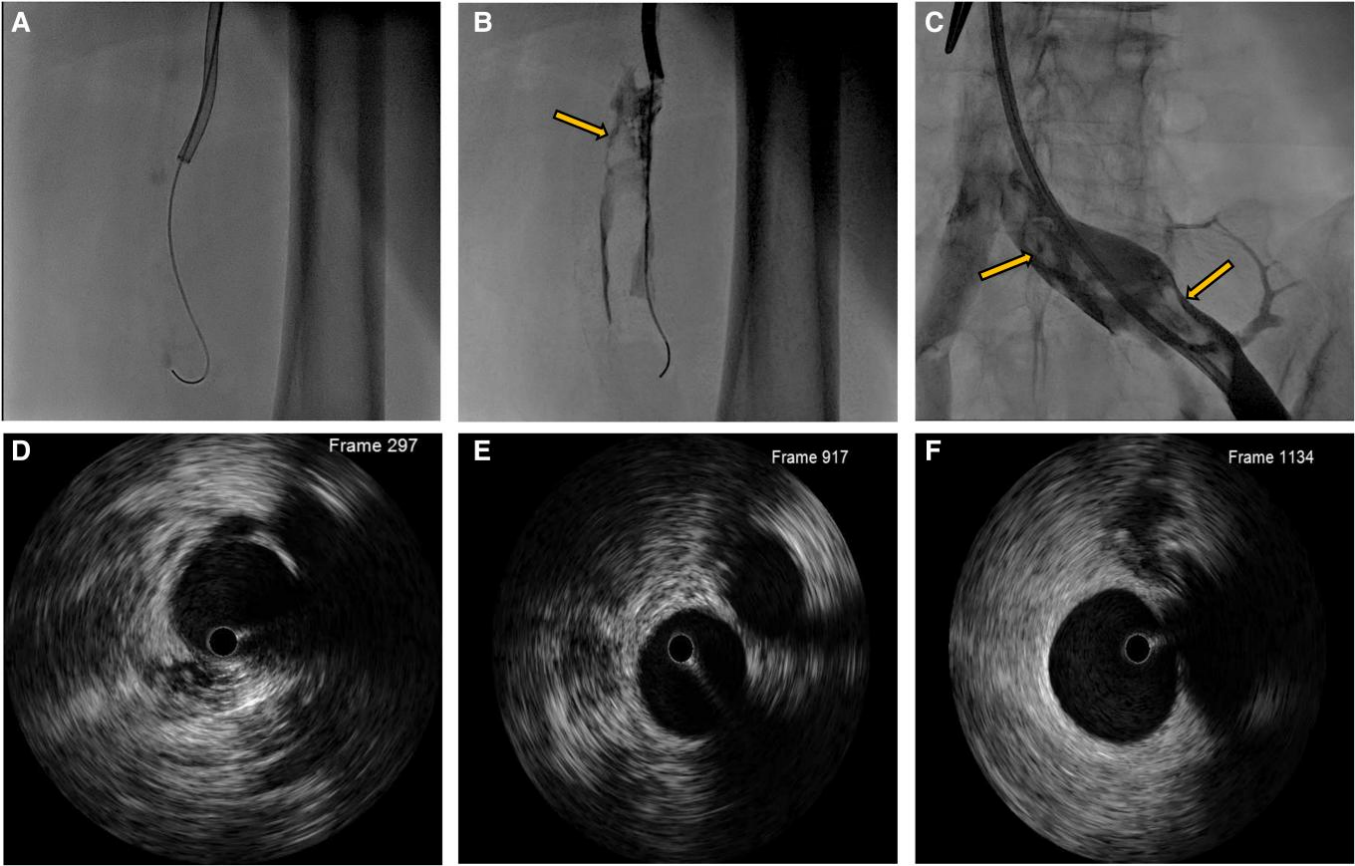
Iliofemoral–popliteal thrombosis and intravascular ultrasound assessment after thromboaspiration. (*A–C*) Contrast venography of the left iliofemoral venous system, highlighting extensive thrombosis. (*D–F*) Intravascular ultrasound images of the popliteal, femoral, and iliac veins following mechanical thromboaspiration, confirming complete resolution of the thrombotic burden and restored vessel patency.

A 74-year-old female with a history of hypertension and diabetes presented to the emergency department of our institution after a syncope at rest resulting in cranial trauma. Ten days prior to admission, she experienced left lower limb swelling, effort dyspnoea, and asthenia without fever or chest pain. At admission, the patient was alert. Initial vitals included blood pressure of 140/95 mmHg, heart rate of 160 b.p.m., and oxygen saturation of 94% on room air. Physical examination revealed clear lung sounds and left lower limb oedema. Arterial blood gas analysis showed a pO₂ of 64 mmHg, pCO₂ of 27 mmHg, and lactate of 2.15 mmol/L. Laboratory tests revealed markedly elevated D-dimer levels (>9000 µg/L) and out-of-range hs-T-troponin of 37.6 pg/mL (n.v. < 14 pg/mL). Electrocardiogram (ECG) showed newly diagnosed atrial fibrillation with rapid ventricular response. Brain computed tomography (CT) scan was performed because cranial trauma and excluded active bleeding. Because of the high suspicion of acute PE, a CT pulmonary angiography (CTPA) was performed and revealed extensive bilateral segmental and subsegmental pulmonary artery thrombi with right atrial and ventricular enlargement and evidence of floating RAT. Lower extremity ultrasound scan showed an extensive DVT in the left ilio-femoro-popliteal axis with a hypo-isoechoic thrombus. Transthoracic echocardiography (TTE) demonstrated significant RV dilatation and dysfunction with left ventricular unloading and confirmed the presence of floating RAT (*Figure 1*). The patient’s clinical course abruptly deteriorated, with blood pressure falling to 80/60 mmHg and lactate levels rising above 2 mmol/L. The presence of floating RAT, alongside patient’s haemodynamic instability, prompted the initiation of ST with recombinant tissue plasminogen activator (rtPA). A 6 F introducer sheath was positioned in the right internal jugular (IJ) vein in order to perform thrombolysis. A 20 mg bolus was followed by an 80 mg infusion over 2 h, resulting in haemodynamic improvement with resolution of shock status and lactate clearance, supported by low-dose dobutamine administration in response to echocardiographic evidence of RV dysfunction. The patient was subsequently transferred to the cardiac intensive care unit (ICU). Despite initial treatment, the persistent floating RAT required a multidisciplinary team discussion involving the PERT, composed of an interventional cardiologist, a vascular surgeon, and an intensive care cardiologist. Given the imminent risk of embolization related to the persistence of floating RAT, emergent percutaneous approach by means of mechanical thrombectomy was indicated. The procedure was performed on a sedated and intubated patient with cardio-anaesthetic support. Intraoperative transoesophageal echocardiography (TOE) confirmed the presence of a large floating thrombus in the right atrium (see *Video 1*). The right IJ vein sheath was changed with a 12 F introducer sheath to guarantee a more direct access to RA. Selective pulmonary angiography was performed using a 5 F Pigtail catheter, showing no residual filling defects, confirming the efficacy of ST and patient’s haemodynamic improvement (see *Video 2*). Under TOE and fluoroscopic guidance, mechanical thromboaspiration was performed using the Penumbra Indigo Lightning 12 F system running on a 0.032 Storq guidewire to avoid direct contact of the suction cannula with the atrial wall and the risk of perforation (see *Figure 2* and *Video 3*). The procedure was effective in solving floating RAT, with no echocardiographic evidence of residual thromboembolic material and decrease in mean pulmonary pressure from 62 to 45 mmHg. Given the extensive proximal DVT, constituting a potential source of PE recurrence, intravascular ultrasound (IVUS) using Opticross 0.035 (Boston Scientific) confirmed preprocedural US findings showing complete ilio-femoro-popliteal DVT, and selective venography of the left iliofemoral axis was performed confirming extensive filling defects in the iliac and femoral veins (see *Figure 3A–C* and *Video 4*). Subsequently, mechanical thromboaspiration with the Penumbra Indigo Lightning 12 F system on a 0.032 Storq guidewire was performed, achieving complete resolution of the thrombosis, confirmed by IVUS control. Intravascular ultrasound also excluded the May–Thurner compression (iliocaval compression) (see *Figure 3D–F*). A total of 350 cc of blood was aspirated with the Penumbra device, and partially organized thrombus was collected in the Penumbra basket. Post-procedure synchronized electrical cardioversion restored sinus rhythm (200 J, two attempts). For intraprocedural hypotension, the patient required norepinephrine and dobutamine support, which were gradually weaned off. Anticoagulation was initiated with unfractionated heparin and transitioned to apixaban. The postoperative course was notable for mild anaemia, requiring transfusion of one unit of packed red blood cells. After exclusion of the May–Thurner syndrome (MTS), further investigations were undertaken to identify potential underlying causes of venous thromboembolism (VTE). A thoracoabdominal CT scan was performed to rule out occult malignancies, while thrombophilia screening and autoimmune panel testing were also completed, both yielding negative results. Although no major provoking factors were identified according to current guidelines, the patient’s profile—characterized by obesity and reduced mobility—was considered a relevant predisposing condition for VTE. Pre-discharge TTE revealed improved RV function with mild residual dilation (basal diameter 50 mm, mid-diameter 37 mm) and a pulmonary artery systolic pressure of 30 mmHg (see *Video 5*). Lower extremity ultrasound scan showed no residual thrombosis in the iliofemoral-popliteal axis. The patient was discharged 10 days post-admission in stable condition, haemodynamically compensated, and maintaining sinus rhythm. At 1- and 3-month follow-ups, she showed full recovery of RV function, resolution of pulmonary hypertension, and no signs of post-thrombotic syndrome in the iliofemoral segment.

## Discussion

High-risk PE is associated with short-term mortality rate exceeding 30%, and ST remains the mainstay treatment unless contraindicated. However, ST failure is reported in a significant proportion of patients with the persistence of haemodynamic instability or the presence of high-risk features such as floating RAT. The management of these clinical conditions remains unclear. Previous studies compared repeated ST with surgical thrombectomy, favouring the latter.^6^ Despite the high risks associated with this strategy, contemporary ESC Guidelines recommend rescue surgical thrombectomy with a higher class of recommendation compared to CDTs, for which data are still lacking. Catheter-directed thrombolysis and mechanical thrombectomy or thromboaspiration have gained increasing traction due to their efficacy in normalizing right heart strain and preventing haemodynamic deterioration in high-risk and intermediate-high risk PE patients and safety in reducing bleeding risk when compared to ST.^7,14^ At the best of our knowledge, CDTs have been studied mainly as a first-choice treatment in high-risk PE but really underused as rescue therapy, which remains a grey zone with lack of data.

We described a clinical scenario in whom ST was effective in restoring haemodynamic stability but with the persistence of floating RAT, a high-risk feature of possible PE recurrence associated with high mortality rate. The Penumbra Indigo Lightning System was effective in solving RAT, like large-bore catheters as reported in literature, and safe, thanks to its small bore and good steerability with a reduced risk of right atrial thin-wall injury, also associated with the support of a 0.035′′ guidewire avoiding the contact between the device and the atrial wall. Our case also highlighted the game-changing role of the PERT in PE management within the broader context of VTE, involving not only the heart and central circulation but also the peripheral vasculature. This multidisciplinary approach enabled the treatment of both floating RAT thrombi and extensive DVT in the same session. Deep vein thrombosis is a known predictor of recurrent PE within the first month and a negative prognostic factor for overall patient outcomes. At the best of our knowledge, this is the first documented case of a combined single-session percutaneous treatment of floating RAT and DVT. Collaboration with vascular surgeon is also essential for the application of intravascular imaging, where IVUS was employed to exclude iliac vein compression by arterial structures against bony landmarks in the iliocaval region, commonly known as the MTS. The MTS is an underrecognized cause of idiopathic DVT, implicated in 2%–5% of patients presenting with symptomatic lower extremity venous disorders.^15^ Intravascular ultrasound is increasingly recognized as the diagnostic gold standard for MTS, allowing precise identification of compressed regions and intraluminal changes in affected vessels.^16,17^

## Conclusion

At the best of our knowledge, our case represents the first single-session, combined percutaneous aspiration of RAT and DVT treatment in a scenario where ST successfully resolved obstructive shock but failed to eliminate high-risk features such as floating RAT. Our case highlights the feasibility and effectiveness of a multidisciplinary approach represented by the PERT model, where interventional cardiologists and vascular surgeons collaboratively performed a combined procedure in a single session. This innovative strategy not only addressed the immediate cause of haemodynamic instability but also reduced the risk of recurrent in-transit thrombi and minimized the likelihood of post-thrombotic syndrome. Unlike previous reports utilizing large-bore aspiration devices, our case employed the Penumbra Indigo Lightning System® as an RAT debulking tool. Its smaller shaft size provided enhanced steerability within the right atrium, reducing the risk of atrial wall injury while maintaining procedural efficacy. Furthermore, the integration of intravascular imaging, such as IVUS, proved instrumental in confirming complete thrombus removal and identifying underlying anatomical contributors such as the MTS, ultimately optimizing patient outcomes.

## Lead author biography


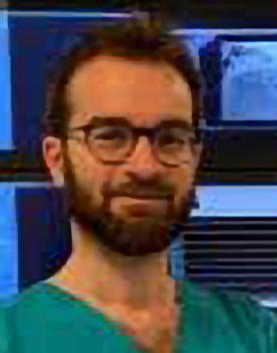


Dr Filippo Russo earned his medical degree and completed his specialization in cardiovascular diseases at the University of Pavia (Italy). He currently serves as an interventional cardiologist at IRCCS San Raffaele Hospital in Milan, Italy. He has developed significant experience in the percutaneous treatment of PE and actively contributes to scientific research and the advancement of technologies in this growing field.

## Data Availability

The data underlying this article will be shared on reasonable request to the corresponding author.

## References

[1] Konstantinides SV, Meyer G, Becattini C, Bueno H, Geersing G-J, Harjola V-P, et al 2019 ESC guidelines for the diagnosis and management of acute pulmonary embolism developed in collaboration with the European Respiratory Society (ERS). Eur Heart J 2020;41:543–603. 31504429 10.1093/eurheartj/ehz405

[2] Koć M, Kostrubiec M, Elikowski W, Meneveau N, Lankeit M, Grifoni S, et al Outcome of patients with right heart thrombi: the right heart thrombi European Registry. Eur Respir J 2016;47:869–875. 26797032 10.1183/13993003.00819-2015

[3] Barrios D, Rosa-Salazar V, Jiménez D, Morillo R, Muriel A, Del Toro J, et al Right heart thrombi in pulmonary embolism. Eur Respir J 2016;48:1377–1385. 27799388 10.1183/13993003.01044-2016

[4] Barrios D, Rosa-Salazar V, Morillo R, Nieto R, Fernández S, Zamorano JL, et al Prognostic significance of right heart thrombi in patients with acute symptomatic pulmonary embolism. Chest 2017;151:409–416. 27746202 10.1016/j.chest.2016.09.038

[5] Marti C, John G, Konstantinides S, Combescure C, Sanchez O, Lankeit M, et al Systemic thrombolytic therapy for acute pulmonary embolism: a systematic review and meta-analysis. Eur Heart J 2015;36:605–614. 24917641 10.1093/eurheartj/ehu218 PMC4352209

[6] Meneveau N, Seronde M-F, Blonde M-C, Legalery P, Didier-Petit K, Briand F, et al Management of unsuccessful thrombolysis in acute massive pulmonary embolism. Chest 2006;129:1043–1050. 16608956 10.1378/chest.129.4.1043

[7] Götzinger F, Lauder L, Sharp ASP, Lang IM, Rosenkranz S, Konstantinides S, et al Interventional therapies for pulmonary embolism. Nat Rev Cardiol 2023;20:670–684. 37173409 10.1038/s41569-023-00876-0 PMC10180624

[8] Pandya YK, Tzeng E. Mechanical thrombectomy devices for the management of pulmonary embolism. JVS Vasc Insights 2024;2:100053. 39749259 10.1016/j.jvsvi.2024.100053 PMC11695062

[9] Pruszczyk P, Klok FK, Kucher N, Roik M, Meneveau N, Sharp AS, et al Percutaneous treatment options for acute pulmonary embolism: a clinical consensus statement by the ESC Working Group on Pulmonary Circulation and Right Ventricular Function and the European Association of Percutaneous Cardiovascular Interventions. EuroIntervention 2022;18:e623–e638. 36112184 10.4244/EIJ-D-22-00246 PMC10241264

[10] Sista AK, Horowitz JM, Tapson VF, Rosenberg M, Elder MD, Schiro BJ, et al Indigo aspiration system for treatment of pulmonary embolism. JACC Cardiovasc Interv 2021;14:319–329. 33454291 10.1016/j.jcin.2020.09.053

[11] Sedhom R, Abdelmaseeh P, Haroun M, Megaly M, Narayanan MA, Syed M, et al Complications of penumbra indigo aspiration device in pulmonary embolism: insights from MAUDE database. Cardiovasc Revasc Med 2022;39:97–100. 34706845 10.1016/j.carrev.2021.10.009

[12] Rosovsky R, Chang Y, Rosenfield K, Channick R, Jaff MR, Weinberg I, et al Changes in treatment and outcomes after creation of a Pulmonary Embolism Response Team (PERT), a 10-year analysis. J Thromb Thrombolysis 2019;47:31–40. 30242551 10.1007/s11239-018-1737-8

[13] Xenos ES, Davis GA, He Q, Green A, Smyth SS. The implementation of a pulmonary embolism response team in the management of intermediate- or high-risk pulmonary embolism. J Vasc Surg Venous Lymphat Disord 2019;7:493–500. 30930079 10.1016/j.jvsv.2018.11.014

[14] Zuin M, Lang I, Chopard R, Sharp ASP, Byrne RA, Rigatelli G, et al Innovation in catheter-directed therapy for intermediate-high-risk and high-risk pulmonary embolism. JACC Cardiovasc Interv 2024;17:2259–2273. 39415385 10.1016/j.jcin.2024.07.033

[15] Tran LM, Go C, Zaghloul M, Malak OA, Hager E, Eslami MH, et al Intravascular ultrasound evaluation during iliofemoral venous stenting is associated with improved midterm patency outcomes. J Vasc Surg Venous Lymphat Disord 2022;10:1294–1303. 35872140 10.1016/j.jvsv.2022.05.016

[16] Gagne PJ, Tahara RW, Fastabend CP, Dzieciuchowicz L, Marston W, Vedantham S, et al Venography versus intravascular ultrasound for diagnosing and treating iliofemoral vein obstruction. J Vasc Surg Venous Lymphat Disord 2017;5:678–687. 28818221 10.1016/j.jvsv.2017.04.007

[17] Secemsky ES, Parikh SP, Kohi M, Lichtenberg M, Meissner M, Varcoe R, et al Intravascular ultrasound guidance for lower extremity arterial and venous interventions. EuroIntervention 2022;18:598–608. 35438078 10.4244/EIJ-D-21-00898 PMC10331977

